# Breast Cancer and Dietary Fat Intake: A correlational study

**DOI:** 10.3126/nje.v9i4.26961

**Published:** 2019-12-31

**Authors:** Preetha J Shetty, Jayadevan Sreedharan

**Affiliations:** 1 Associate Professor, Department of Biomedical Sciences, College of Medicine, Gulf Medical University, Ajman, United Arab Emirates; 2 Professor, Department of Community Medicine, College of Medicine, Gulf Medical University, Ajman, United Arab Emirates

**Keywords:** Breast cancer, Obesity, fat consumption

## Abstract

**Background:**

Breast cancer (BC) is reported to be the most common malignancies affecting women worldwide. There is a sharp increase in the BC incidence rate over the past three decades as previously low risk countries are moving towards high -calorie western diets. Few epidemiologic studies along with animal experiments have ascertained the role of dietary fat in developing BC. This study aimed to determine the correlation between per capita dietary fat consumption and incidence of BC.

**Methods:**

Three major data bases were used to conduct this correlational study. The data regarding consumption of fat and breast cancer incidence from 88 countries across five continents were extracted. The correlation coefficients between the incidence of BC and the fat consumption from the year 1990 to 2007 were calculated.

**Results:**

A statistically significant (P<0.001) correlation between the average fat consumption and the crude BC incidence rate was observed and was more than 0.6, clearly indicating that there is a moderate to strong correlation with fat consumption and incidence of breast cancer (P<0.001).

**Conclusions:**

Our observation indicated that increased total fat consumption increases the risk of developing BC. Consumption of dietary fat increases obesity thereby, increasing the risk of BC development. Dietary fat gets stored in the body since they undergo minimum oxidation as compared to carbohydrates and protein thereby, contributing to obesity a known risk factors for BC. Current study strengthens the evidence to support the hypothesis that non-genetic factors contribute to the occurrence of this disease.

## Introduction

As per the International Agency for Research on Cancer (IARC), BC is reported to be the most common malignancies affecting women worldwide [1]. Due to its psychological impact undoubtedly, it is the most-feared cancer in women affecting the perception of sexuality and self-image significantly than any other cancer [2]. Breast cancer is successfully treatable if detected early [3], but morbidity and mortality due to it is still very high.

BC incidence rates in developed countries are approximately 90-130 per 100,000 women causing 15.4% of total cancer deaths in these countries and are approximately 10-60 per 100,000 women in developing countries causing 14.3% of total cancer deaths in these countries [4]. The trend shows that the incidence of breast cancer is increasing sharply since past three decades in low risk countries including developing Asian countries [5,6]. Recent studies indicate that BC incidence is increasing in developed countries as it is moving towards high-calorie western diets [7-9].

There is a slow progressive increase in incidence of BC, while the mortality due to this malignancy has been stable. The maintenance of stable mortality rate in the face of a rising incidence despite of early diagnosis due to screening programs is a source of concern. There are numerous factors which are considered to contribute to the disease etiology, which vary with respect to area, community, socio-economic conditions, life-styles etc. Race, parity and life-style related problems might be associated with breast cancer [1].

Several studies from around the world have identified the factors associated with the occurrence of breast cancer [10-13]. Advanced age at first childbirth and nulliparity are the most consistent risk factors associated with BC [14]. High energy rich diet such as fat and meat also plays a major role in causing BC [15-17].

Few epidemiological studies and animal experiments have supported the hypothesis that dietary fat has the potential role in the occurrence of BC [17-19]. Case-control studies observed that consumption of total fat and specific type of fat is associated with BC among adult women [20-23]. Women who consumed high level of fat had 13% increased risk of developing BC as per the evidence provided by a meta-analysis [17]. A study by Binukumar et al., reported that estrogen level is a main determinant for BC; dietary fat increases the production of endogenous estrogen thereby leading to BC [24]. The real association between fat intake and breast cancer risk is not still consistent. The aim of the present correlational study is to determine the correlation between per capita dietary fat consumption and incidence of breast cancer.

## Methodology

The correlation coefficients between the incidence of breast cancer for the years 1995, 2000, 2001, 2002, 2003, 2004, 2005, 2006 and 2007 and the fat consumption for the years 1990-92, 2000-02 and 2005-07 were calculated.

Ethics: Ethical approval was not taken as it is a correlational study using various data sets available in the literature. The data bases used were with the no copyright.

Statistics: Spearman rank correlation was used to determine degree of relation between the fat consumption and the incidence of breast cancer.

## Results

This study observed a statistically significant (P<0.001) correlation between the average fat consumption and the crude breast cancer incidence rate. First, we have calculated the correlation coefficient between the average fat consumption during the year 1990-92 and the breast cancer incidence during 1995, 2000, 2001, 2001, 2003, 2004, 2005, 2006 and 2007. All the years the correlation coefficient observed was more than 0.6, which showed there is a moderate to strong correlation with fat consumption and incidence of breast cancer [Table 1].

The correlation coefficient between age standardized incidence rate and the fat consumption is given in table 2. All the places we observed a statistically significant (P<0.001) strong positive correlation.

The fat consumption during the year 1990-92 is correlated with the breast cancer incidence rate of 1995, 2000, 2001, 2002, 2003, 2004, 2005, 2006 and 2007. The correlation coefficient was very high (0.89) with 1990-92 fat consumption and 1995 cancer incidence rate. Similarly, for the average fat consumption during 1995-97 is correlated with 2000 to 2007 as it not ideal to correlate with 1995 cancer incidence. All the years, the coefficient was almost same (>0.80). For the fat consumption of 2000-02 is correlated with 2001-2007 and 2005-07 fat consumption data is correlated with only the year 2006 and 2007 [Table 2].

## Discussion and way forward:

Studies till date clearly indicate that there is an association between fat consumption and occurrence of BC. As the consumption increases the chances of developing this disease also increases [20,21,29]. However, few studies have concluded that there is no association between fat consumption and breast cancer development [13,30]. A study from Japan by Hirayama et al. reported that high amount of meat consumption on a daily basis led to increase the incidence of BC among women [31]. There are certain limitations in measuring the amount of fat intake using food frequency-based questionnaires which are used in case-control and cohort studies.

The current study is a correlational study in which three major databases were used to extract information on per capita fat consumption and BC incidence. The observations are abstracted from the databases which aim to specifically measure the fat consumption and incidence of BC. Hence the data is more reliable and precise. This study clearly indicates a statistically significant (p<0.001) correlation between the average fat consumption and the crude breast cancer incidence rate. Consumption of dietary fat increase obesity thereby increasing the risk of breast cancer development. Since the dietary fat is more efficiently stored in the body for the simple fact that fat influences its own oxidation weakly or not at all as compared to carbohydrates and proteins which promote their own oxidation [32-34].

## Conclusion:

The evidence from the current study strengthens the hypothesis that non-genetic factors like dietary fat plays an important role in the causation of BC. In view of epidemiologic evidence, it can likely be concluded that a positive correlation exists between breast cancer incidence and the total dietary fat intake. A study by Bjarnason et al. among the Icelandic population proved that higher fat composition over a period causes an increase in the incidence of breast cancer [35]. However, there are studies which do not agree with the direct correlation between the two. The factors leading to the discrepancy in study findings may be because of the methodology in measuring the diet intake and other confounders such as energy intake, physical activity, age and cigarette smoking [36,37].

## Figures and Tables

**Table 1:** The Correlation between fat consumption and crude breast cancer incidence rate

Fat consumption during	Correlation and significance	Crude breast cancer incidence rate								
		1995	2000	2001	2002	2003	2004	2005	2006	2007
**1990-1992**	Correlation Coefficient	0.79	0.64	0.64	0.64	0.67	0.66	0.62	0.62	0.61
	Sig. (2-tailed)	<0.001	<0.001	<0.001	<0.001	<0.001	<0.001	<0.001	<0.001	<0.001
	N	57	127	127	127	127	127	127	126	125
**1995-1997**	Correlation Coefficient	--	0.67	0.67	0.68	0.72	0.70	0.69	0.68	0.66
	Sig. (2-tailed)	--	<0.001	<0.001	<0.001	<0.001	<0.001	<0.001	<0.001	<0.001
	N	--	127	127	127	127	127	127	126	125
**2000-2002**	Correlation Coefficient	--	--	0.63	0.63	0.66	0.65	0.62	0.61	0.61
	Sig. (2-tailed)	--	--	<0.001	<0.001	<0.001	<0.001	<0.001	<0.001	<0.001
	N	--	--	127	127	127	127	127	126	125
**2005-2007**	Correlation Coefficient	--	--	--	--	--	--	--	0.56	0.56
	Sig. (2-tailed)	--	--	--	--	--	--	--	<0.001	<0.001
	N	--	--	--	--	--	--	--	126	125

**Table 2:** The Correlation between fat consumption and age standardized breast cancer incidence rate

Fat consumption during	Correlation and significance	Age standardized breast cancer incidence rate								
		1995	2000	2001	2002	2003	2004	2005	2006	2007
**1990-1992**	Correlation Coefficient	0.89	0.81	0.83	0.83	0.84	0.84	0.81	0.82	0.83
	Sig. (2-tailed)	<0.001	<0.001	<0.001	<0.001	<0.001	<0.001	<0.001	<0.001	<0.001
	N	57	127	127	127	127	127	127	126	125
**1995-1997**	Correlation Coefficient	--	0.80	0.81	0.82	0.83	0.82	0.80	0.81	0.82
	Sig. (2-tailed)	--	<0.001	<0.001	<0.001	<0.001	<0.001	<0.001	<0.001	<0.001
	N	--	127	127	127	127	127	127	126	125
**2000-2002**	Correlation Coefficient	--	--	0.82	0.83	0.83	0.83	0.81	0.82	0.83
	Sig. (2-tailed)	--	--	<0.001	<0.001	<0.001	<0.001	<0.001	<0.001	<0.001
	N	--	--	127	127	127	127	127	126	125
**2005-2007**	Correlation Coefficient	--	--	--	--	--	--	--	0.82	0.83
	Sig. (2-tailed)	--	--	--	--	--	--	--	<0.001	<0.001
	N	--	--	--	--	--	--	--	126	125
